# Sperm motility assessed by deep convolutional neural networks into WHO categories

**DOI:** 10.1038/s41598-023-41871-2

**Published:** 2023-09-07

**Authors:** Trine B. Haugen, Oliwia Witczak, Steven A. Hicks, Lars Björndahl, Jorunn M. Andersen, Michael A. Riegler

**Affiliations:** 1 Department of Life Sciences and Health, OsloMet – Oslo Metropolitan University, Oslo, Norway; 2 Simula Metropolitan Center for Digital Engineering, Oslo, Norway; 3 ANOVA, Karolinska University Hospital and Karolinska Institutet, Stockholm, Sweden

**Keywords:** Cell biology, Computational biology and bioinformatics, Medical research

## Abstract

Semen analysis is central in infertility investigation. Manual assessment of sperm motility according to the WHO recommendations is the golden standard, and extensive training is a requirement for accurate and reproducible results. Deep convolutional neural networks (DCNN) are especially suitable for image classification. In this study, we evaluated the performance of the DCNN ResNet-50 in predicting the proportion of sperm in the WHO motility categories. Two models were evaluated using tenfold cross-validation with 65 video recordings of wet semen preparations from an external quality assessment programme for semen analysis. The corresponding manually assessed data was obtained from several of the reference laboratories, and the mean values were used for training of the DCNN models. One model was trained to predict the three categories progressive motility, non-progressive motility, and immotile spermatozoa. Another model was used in predicting four categories, where progressive motility was differentiated into rapid and slow. The resulting average mean absolute error (MAE) was 0.05 and 0.07, and the average ZeroR baseline was 0.09 and 0.10 for the three-category and the four-category model, respectively. Manual and DCNN-predicted motility was compared by Pearson’s correlation coefficient and by difference plots. The strongest correlation between the mean manually assessed values and DCNN-predicted motility was observed for % progressively motile spermatozoa (Pearson’s r = 0.88, p < 0.001) and % immotile spermatozoa (r = 0.89, p < 0.001). For rapid progressive motility, the correlation was moderate (Pearson’s r = 0.673, p < 0.001). The median difference between manual and predicted progressive motility was 0 and 2 for immotile spermatozoa. The largest bias was observed at high and low percentages of progressive and immotile spermatozoa. The DCNN-predicted value was within the range of the interlaboratory variation of the results for most of the samples. In conclusion, DCNN models were able to predict the proportion of spermatozoa into the WHO motility categories with significantly lower error than the baseline. The best correlation between the manual and the DCNN-predicted motility values was found for the categories progressive and immotile. Of note, there was considerable variation between the mean motility values obtained for each category by the reference laboratories, especially for rapid progressive motility, which impacts the training of the DCNN models.

## Introduction

A basic semen analysis is central in infertility investigation and implies examinations of sperm number, sperm motility, and sperm morphology^1^. Manual assessment of these variables according to WHO recommendations is regarded as the gold standard^1,2^. This standardization is essential in clinical work, for comparability across laboratories and over time, and for the establishment of reference limits for semen parameters^1,3,4^. For sperm motility, it includes to estimate the proportion of spermatozoa in different categories of motility, or as immotile spermatozoa. However, the manual motility assessment is time-consuming and requires comprehensive training, which is a prerequisite to obtain accurate and reproducible results.

In recent years, computer-aided sperm analysis (CASA) systems have been improved, however, these systems still have limitations in analyzing undiluted human ejaculates^1,5,6^. Moreover, the various CASA systems differ in technology, and the result of the analyses will be instrument-dependent. CASA may be valuable in identifying characteristics of spermatozoa prepared for medically assisted reproduction, but is not recommended in routine assessment^1^.

The potential application of artificial intelligence in reproductive medicine has resulted in increased research activity to develop algorithms for use in semen analysis^7,8^. Most of the work has focused on sperm morphology to improve the accuracy of the assessment and make the classification automated. There have also been several approaches to developing machine learning models for tracking sperm movement, however, few studies have investigated how algorithms are able to predict sperm motility^9–14^.

Within the field of machine learning, deep learning is the current state of the art. Deep convolutional neural networks (DCNNs) are especially suitable for image classification and may therefore be applicable in assessing sperm motility. DCNNs learn the important features automatically from the data. These features might not make sense to humans, but they can contain important information to solve the task at hand. This can also allow us to discover new knowledge through model explanations that describe what information the DCNN uses to make a specific prediction.

However, the algorithms need training data and can only perform as well as the quality of the provided data permits. To develop DCNN models with high predictive power for sperm motility categorization, the dataset used should be based on accurate and reproducible methods and of a quality as good as possible in terms of image resolution and ground truth data.

We have previously investigated the performance of various machine learning models in analyzing sperm motility from videos of fresh semen samples^14^. The data in that study was based on manual assessment of three categories of motility as described in WHO 2010 Manual^15^, performed by one person. This dataset has also been used in two recent studies^12,13^. According to the WHO recommendations from 2010, the assessment of sperm motility is based on a system that distinguishes motile spermatozoa from immotile and progressive motility from non-progressive. We found that the DCCN model ResNet-50 overall performed best. In the most recent recommendations from WHO^1^, the differentiation of progressive motility into rapid and slow was again recommended, as in the WHO 1999 manual^16^.

The aim of the present study was to test ResNet-50 architecture on sperm motility videos obtained from an external quality assessment programme for semen analysis, using the corresponding manual data provided by several reference laboratories participating in the programme. The assessment did also include the differentiation of progressive motility into rapid and slow. The performance of the DCNN models in predicting the proportion of spermatozoa into the motility categories was compared with the manual assessments. The evaluation of each sample was performed on the same clips of the videos of wet semen preparations.

## Materials and methods

### Dataset

Videos of wet preparations (400 × magnification) of 65 fresh semen samples were obtained from the ESHRE-SIGA EQA Programme for Semen Analysis between 2006 and 2018 and used in the development of the DCNN model. All participating laboratories analyzed the same videos and sent their results back to the EQA programme manager after analysis. The video clips were assessed by personnel from four to ten laboratories. Data was stored and shared for comparison between labs. The dataset used for training the DCNN models contains these videos and mean values of motility results provided by the reference laboratories. The dataset used to train the models in this study is openly available and can be downloaded at^17^.

**Figure 1:**
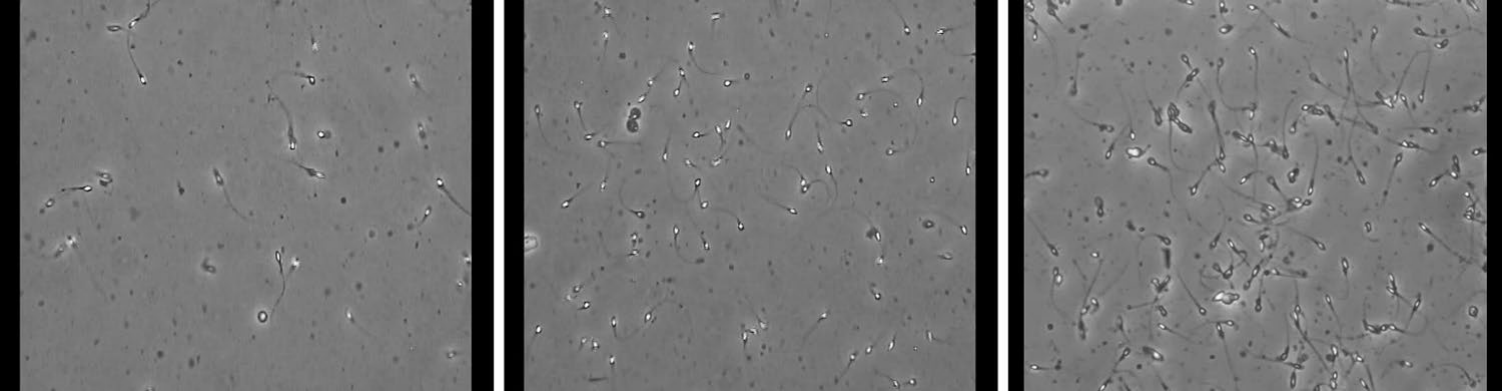
Example frames from three different videos from the presented dataset.

The videos were recorded at the Andrology Unit, Karolinska University Hospital, and Karolinska Institutet, and distributed among the participating laboratories in the programme. The ejaculates were incubated at 37 °C, and the videos were made 30–60 min after collection. The temperature during the recordings was kept at 37 °C by use of pre-heated slides placed on microscope trays with a controlled temperature of 37 °C. Randomly chosen fields were recorded for 5–10 s, depending on the number of visible spermatozoa. The number of fields was chosen to allow the assessment of at least 200 spermatozoa. Some of the investigators used a mask with a 25 × 25 um reticle to facilitate the assessment of sperm motility. Micrometer scale recorded at the beginning of each DVD, with the same magnification as the actual sample recording, allowed each investigator to calibrate their own screen used. The manual assessment of the videos was performed according to WHO 1999 criteria^16^. Sperm motility was categorized into rapid progressive (grade a), slow progressive (grade b), non-progressive (grade c), and immotile (grade d). In addition, we calculated the proportion of total progressive motility (a + b), which is a part of the categorization system in the WHO 2010 manual^15^. Examples of frames from videos are shown in Fig. 1.

### Model development

**Figure 2:**
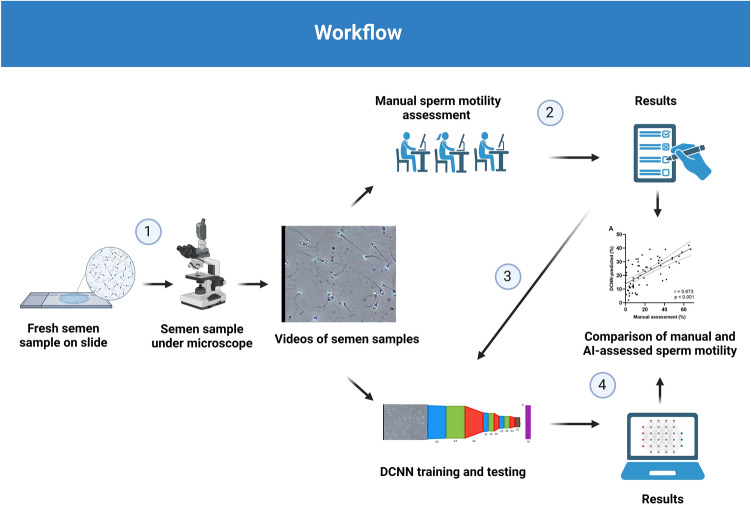
Chart of workflow. (1) Semen samples from 65 individuals were placed on slides, and sperm motility was recorded by a microscope-mounted video camera. (2) Videos were distributed to reference laboratories for manual sperm motility assessment according to WHO recommendations. (3) Videos (n = 65) and mean values (n = 65) from the manual sperm motility assessment were used to train and test the three-category and four-category DCNN models. Optical flow generated from batches of 30 frames collected across the entire length of each video, was used to develop the models. (4) DCNN-predicted values were compared to manual results. The figure was created by the authors with Biorender.com.

Lucas–Kanade optical flow was estimated for every second of the video and then visualized as an image, which was then used as input to the DCNN. In essence, this compresses the temporal information about the movements of the spermatozoa into a single image that can more easily be interpreted by the DCNN model. The videos have a framerate of 30 frames per second, making each optical flow-generated image represent motion calculated across 30 frames. The DCNN architecture is based on ResNet-50, which has previously shown promising results when analyzing videos of live spermatozoa^14^. The ResNet-50 is built with a Global Average Pooling layer at the end and trained on optical flow-based images generated by Lucas-Kanade. For training, we used the optimizer Adam with a learning rate of 0.0004 and calculated loss using mean absolute error (MAE). Otherwise, the default parameters included in the Keras Python package were used. For the baseline, ZeroR (pseudo regression) was performed. Results are reported as MAE, where the closer MAE is to 0, the more accurate the model is. Ten-fold cross-validation was used to compensate for the relatively small dataset, where per fold, one-tenth of the data was used as an independent validation dataset and left out from training. The model was trained for a maximum of 1,000 epochs (rounds of training the model), where we stopped training if the model did not improve on the validation dataset for the last 15 epochs. Using the DCNN architecture, we trained two models, one for predicting the percentage of spermatozoa in the motility grades a, b, c, and d, the four-category model, and one for predicting the percentage of spermatozoa in the motility grades a + b, c, and d, the three-category model. An overview of the work is presented in Fig. 2.

### Statistical analysis

Analyze-it for Excel version 5.30.1 and GraphPad Prism version 9.0 were used for statistical analysis and for the generation of plots. Scatter plots were used to visualize the correlations between manual and DCNN-predicted motility. Associations were assessed by Pearson’s correlation coefficient. P-values < 0.01 were considered significant. Difference plots were used to compare manual and DCNN-predicted motility. The bias was estimated as the median of the differences and is shown as a horizontal line, and 95% limits of agreement (LoA) were added in dashed lines to display outliers. Line plots were used to visualize the difference in results between the manual assessment and the results predicted with DCNN. Mean values from the manual assessment were sorted from lowest to highest for the respective categories. Minimum and maximum values were added to indicate the interlaboratory variation for each sample. Minimum and maximum values were available for 43 samples.

## Results

Both DCNN models showed high predictive power. For the three-category model, the MAE was 0.06 for progressive, 0.04 for non-progressive, and 0.05 for immotile spermatozoa, resulting in an average MAE of 0.05. The average three-category ZeroR baseline was 0.09. For the four-category model, the MAE was 0.11 for rapid progressive, 0.09 for slow progressive, 0.04 for non-progressive, and 0.05 for immotile spermatozoa. The average MAE was 0.07, and the average four-category ZeroR baseline was 0.10.

**Figure 3:**
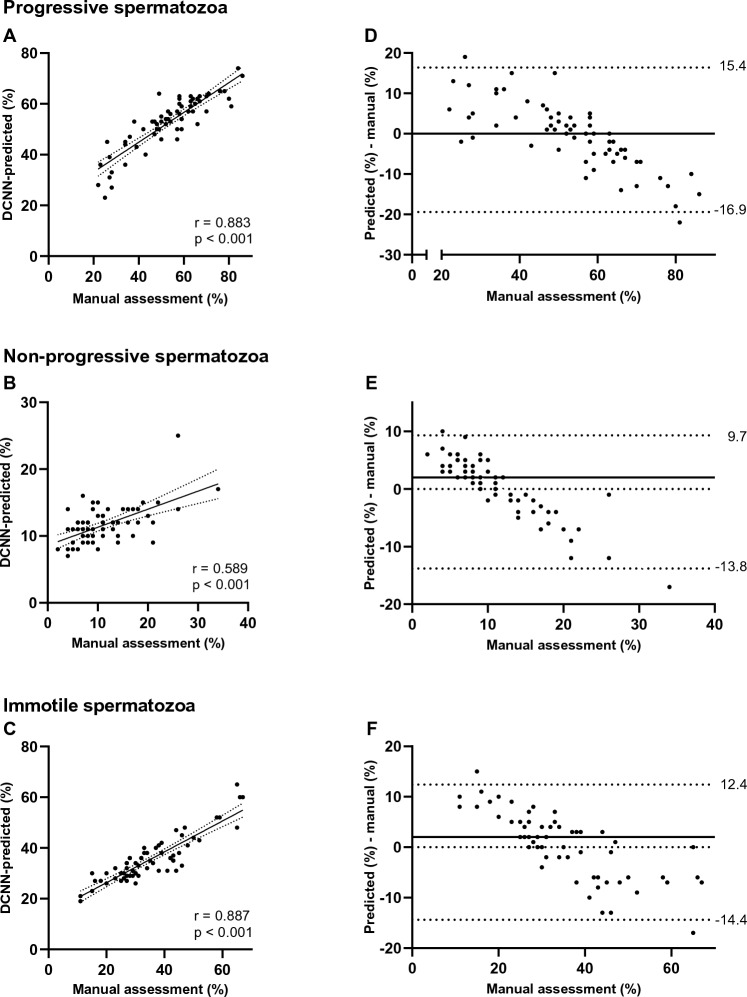
Comparison of manually and DCNN-predicted sperm motility in a three-category model. Correlation graphs (**A**–**C**) and difference plots (**D**,**E**) between sperm motility assessed manually by reference laboratories and motility values predicted for three categories by deep convolutional neural network (DCNN); (**A**) and (**D**) progressive spermatozoa, (**B**) and (**E**) non-progressive spermatozoa, (**C**) and (**F**) immotile spermatozoa. In the correlation graphs, solid lines represent the trendlines while dashed lines the 95% confidence interval. Pearson’s correlation coefficient (r) and p-value (p) between results are also depicted. In the difference plots, the bias estimated as the median of the differences between predicted and manually assessed motility is shown as a solid line, while the 95% limits of agreement (LoA) are represented in the figure and shown in dashed lines to display outliers.

We compared the mean values from the manual assessment with the DCNN-predicted for the three motility categories recommended in the WHO manual from 2010^15^; progressive, non-progressive, and immotile spermatozoa. The correlation between the mean values based on the reference laboratories and DCNN-predicted motility for the various categories is shown in Fig. 3. Strongest correlation was observed for % progressively motile spermatozoa (Pearson’s r = 0.88, p < 0.001) (Fig. 3A) and % immotile spermatozoa (r = 0.89, p < 0.001) (Fig. 3C). At low proportion of progressively motile spermatozoa, the discrepancy between the assessments was most pronounced.

**Figure 4:**
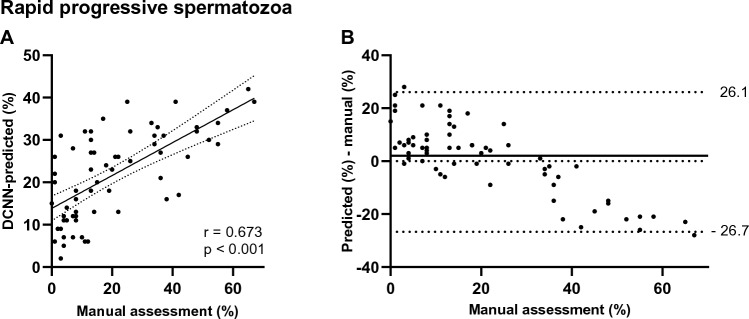
Comparison of manually and DCNN-predicted rapid progressive sperm motility. Correlation graph (**A**) and difference plot (**B**) between rapid progressive motility assessed manually by reference laboratories and motility values predicted for four categories by deep convolutional neural network (DCNN). In the correlation graph, solid line represents the trendlines while dashed lines the 95% confidence interval. Pearson’s correlation coefficient (r) and p-value (p) between results are also depicted. In the difference plot the bias estimated as the median of the differences between predicted and manually assessed motility is shown as a solid line, while the 95% limits of agreement (LoA) are represented in the figure and shown in dashed lines to display outliers. For the results between all the four categories, manually and DCNN-predicted, see Supplementary Fig. S1.

We also included a comparison of the results for rapid progressive motility, which is based on the assessment of four categories; rapid progressive, slow progressive, non-progressive, and immotile spermatozoa^1,16^ (Fig. 4 and Supplementary Fig. S1). The correlation between the manually assessed and the DCNN-predicted rapid progressive motility was moderate (Pearson’s r = 0.673, p < 0.001) (Fig. 4A).

For the three-category model, the median difference (bias) when comparing manual against predicted progressive motility, non-progressive motility, and immotility was 0, 2, and 2, respectively (Fig. 3D–F). The 95% LoA was broader for the progressive category than for the immotile. Furthermore, the predicted results had a tendency to be overestimated in the lower percentage range and underestimated for high percentages, for all the motility categories. These trends were also observed for the four-category model (Supplementary Fig. S1).

**Figure 5:**
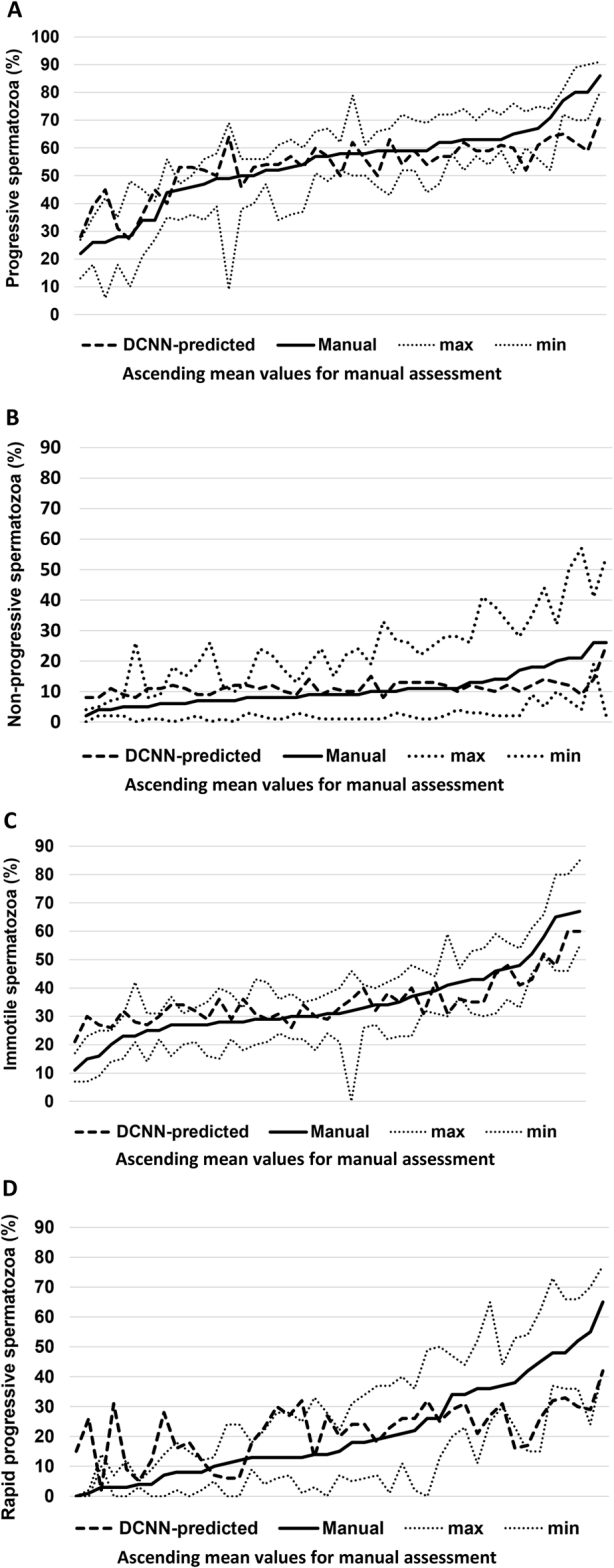
Comparison of manually and DCNN-predicted sperm motility at increasing order of manual mean values for the individual samples. Line plots showing mean, maximum and minimum values (n = 43) from manual assessment and DCNN-predicted values for rapid progressive (**A**), slow progressive (**B**), non-progressive motility (**C**), and immotility (**D**).

Figure 5A–D depicts the manually assessed mean values of progressive, non-progressive, immotile, and rapid progressive spermatozoa for the individual samples in increasing order, as well as the predicted values. The maximum and minimum values for the reference laboratories are also plotted in the figures, showing the discrepancy in the manual assessments. For most of the samples, the DCNN-predicted value was within the range of the interlaboratory variation of the results. The deviation in predicted value was largest at the lower proportion of rapid, non-progressive, and immotile spermatozoa, where an overestimation was observed.

## Discussion

A more objective assessment of sperm motility has been one of the goals in the development of CASA systems, but this has been a challenge, especially with undiluted human semen samples^1,5,6^. Previous studies involving the use of AI for motility assessment have focused on comparing the performance of various models or exploring the sperm kinematics in prepared samples using for example traditional supervised machine learning^9^ and in fresh semen samples applying a Region-Based Convolutional Neural Networks (R-CNN) architecture^10^. The current state-of-the-art method for image and video analysis is DCNNs, which can extract relevant features and utilize them for classification or segmentation. Thus, DCNNs have the potential to assess sperm motility from videos. We have previously demonstrated that deep learning models are able to predict sperm motility better than the classical machine learning algorithms in terms of mean MAE for each motility category in a three-category system^14^. Of the DCNN models used, the ResNet-50 model overall performed best. The manual assessment of sperm motility in that previous study was performed according to WHO recommendations, but by only one person. In the present work, the architecture from the former study was used to train both a three-category and a four-category model based on new data of higher quality. The DCNN models were trained by using the mean of the results from several well-trained personnel at different locations, leading to more reliable models. The earliest data in our study was from 2006, when the recommendations from WHO 1999 were followed^16^. The ESHRE-SIGA External Quality Assessment Programme continued with the four-category system for sperm motility assessment also after the WHO 2010 manual was published^18^. Categorization of progressive motility into rapid and slow was not a part of the recommendation of the WHO 2010 manual, mainly based on the argument that this distinction was difficult to obtain accurately^15^. However, the identification of rapid progressive spermatozoa has been found to be of clinical importance, and the four-category system is again recommended in the latest edition of the manual^1,19–24^.

We found that DCNN worked well in predicting progressive motility and immotility. Among the motility categories, we found the lowest correlation between the manual and the DCNN-predicted values for slow progressive motility. However, when comparing the manual and the predicted values for the individual samples (Fig. 5), it can be seen that there is considerable variation between the reference laboratories, especially for the rapid progressive motility. Thus, the challenge in obtaining accurate assessments for this category is also reflected in the DCNN-predicted results, which are within the range of variation between the reference laboratories for most samples. More data with high-quality and precise annotations will be needed to develop models that perform better. However, from a clinical point of view, the presence of rapid progressive spermatozoa (≥ 25 um/s) is important, although the exact velocities of the rapid spermatozoa are not determined. Determination of the proportion and the total number of rapid spermatozoa would therefore be of clinical interest.

The performance of the DCNN has two main directions for future improvements. Firstly, the quality in terms of visual details and resolution of the provided videos is mid to low level based on present technical standards. In the future, it is important to produce video datasets of higher quality, e.g., of high resolution like 4 k and no artifacts, in combination with assessments of the videos by well-trained staff. Such a dataset could also be used to develop more sophisticated methods. Secondly, for now, we are processing the videos in batches of 30 frames, disregarding any shifts in the video. In the future, a strategy would be to create ensemble networks that can recognize temporal and detailed information from the spermatozoa by providing, for example, segmentation masks that track individual sperm across the entire video. Multimodal input learning data could allow tackling the challenge with multi-instance learning. For example, one task could be to learn the number of spermatozoa and the second one the movement patterns, which in combination could lead to a better overall prediction. This would also be completely different from the current CASA.

The relatively small dataset is a limitation. To compensate for the size, ten-fold cross-validation was used. However, even though cross-validation is acceptable for testing model performance and comparing it to other models on the same dataset^9,25^, it does not test the generalizability of the results. An independent test set evaluation should be performed, preferentially across different clinics^26^.

Moreover, the dataset consists of videos of relatively homogenous samples. There are differences in sperm concentration and motility, but no samples had a high degree of debris, agglutinates, aggregates, or round cells. The consequence of this is that it is unknown how the model will perform with more heterogeneous samples, and more rigorous testing is needed in this regard. However, the results from our previous study indicated that the predictive power of the DCNN model was independent of concentration ranging from 4 × 10^6^/mL to 350 × 10^6^/mL^14^.

A strength of this study is that the same video clips were evaluated by the laboratory personnel and by the DCNN models. Furthermore, the data used for training the DCNN models was based on an assessment performed by several well-trained laboratory personnel. The interlaboratory variation between the reference laboratories, as shown in Fig. 5, reflects the challenge of obtaining reproducible results, even for highly trained personnel. In this respect, a DCNN model is promising for developing a more objective and reproducible method for motility assessment. This is important for the development of accurate and clinically useful techniques to evaluate the functions of the male reproductive organs and male infertility factors. An additional DCNN model could be trained in future work in tracking the spermatozoa to perform more fine-grained analysis (e.g., include distance moved and movement patterns). Although it is clear that AI will not be able to replace manual motility assessment in the near future, support from the AI model for manual evaluation can be beneficial. It is considerably less time-consuming and compared with CASA systems, might also be performed at a low cost. There would still be a need for a manual external and internal quality control programme, but these could be run more effectively with the support of automated systems. For example, one could do a baseline performance analysis using the AI model to quickly identify poor samples. Overall, this could encourage the establishment of a DCNN method in the clinics as the basis for improved diagnosis and management of male infertility, and for examining the semen sample of vasectomized men. A more objective and less resource-demanding tool will also be of value for performing multicentre studies. Finally, it is important to develop AI models for the evaluation of other sperm characteristics to obtain a more objective semen analysis.

## Conclusion

DCNN models performed well in predicting the proportion of spermatozoa in the WHO motility categories, especially for the progressive and immotile spermatozoa. For most samples, the DCNN-predicted value was within the range of the interlaboratory variation. For clinical purposes, a more reliable model for evaluating rapid progressive spermatozoa should be developed. DCNN models could provide a cost- and time-effective method for motility assessment, however, there is still a need for improvement. A prerequisite is larger manually assessed datasets of high quality for training the models. It is crucial to test the DCNN models in studies with data from different clinics and with varying degrees of quality of the samples.

### Supplementary Information

Supplementary Information.

## Data Availability

The datasets generated during and/or analysed during the current study are available at https://datasets.simula.no/visem-qc.
