# Supplementary material for: Sperm motility assessed by deep convolutional neural networks into WHO categories
Source: Sci Rep. 2023 Sep 7;13:14777. doi: 10.1038/s41598-023-41871-2 (PMC10484948; doi:10.1038/s41598-023-41871-2)
Supplement: Supplementary file 1 — Supplementary Information. [file 41598_2023_41871_MOESM1_ESM.pdf]

Sperm motility assessed by deep convolutional neural networks into WHO categories

Trine B. Haugen[1*], Oliwia Witczak[1], Steven A. Hicks[2], Lars Björndahl[3], Jorunn M. Andersen[1§], Michael Riegler [2§]

[1]Department of Life Sciences and Health, OsloMet – Oslo Metropolitan University, Oslo, Norway
[2]Simula Metropolitan Center for Digital Engineering, Oslo, Norway
[3]ANOVA, Karolinska University Hospital and Karolinska Institutet, Stockholm, Sweden

[*]Correspondence: Trine B. Haugen, Department of Life Sciences and Health, OsloMet – Oslo Metropolitan University, Oslo, Norway, tribha@oslomet.no

[§]These authors have contributed equally to this work

Supplementary Figure 1

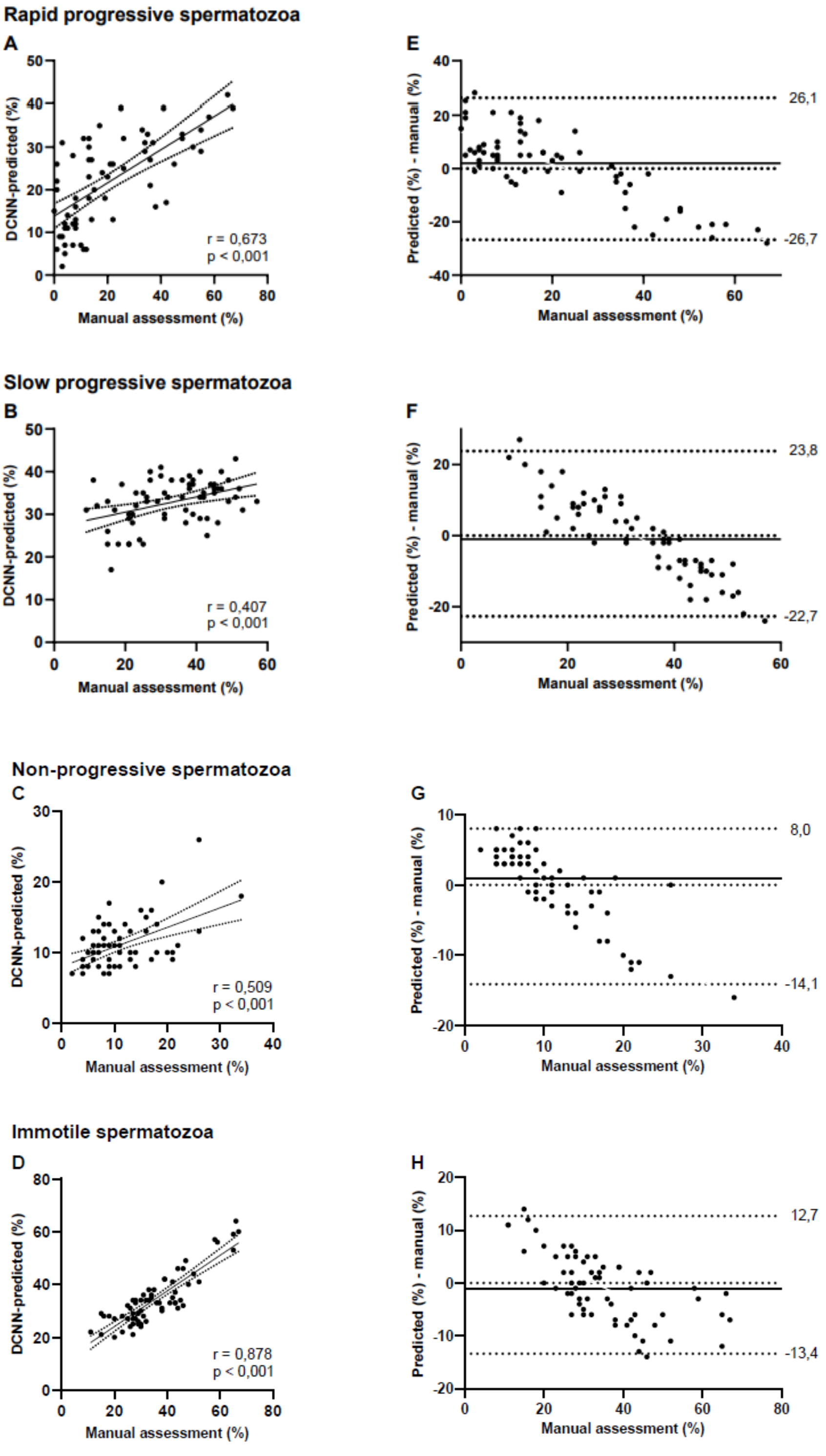

Supplementary Figure S1.

Comparison of manually and DCNN-predicted sperm motility in a four-category model. Correlation graphs (A-D) and difference plots (E-H) between sperm motility assessed manually by reference laboratories and motility values predicted for four categories by deep convolutional neural network (DCNN); (A) and (E) rapid progressive spermatozoa, (B) and (F) slow progressive spermatozoa, (C) and (G) non-progressive spermatozoa, (D) and (H) immotile spermatozoa. In the correlation graphs, solid lines represent the trendlines while dashed lines the 95% confidence interval. Pearson's correlation coefficient (r) and p-value (p) between results are also depicted. In the difference plots, the bias estimated as the median of the differences between predicted and manually assessed motility is shown as a solid line, and the 95% limits of agreement (LoA) are represented in the figure and shown in dashed lines to display outliers.
